# Production of the Main Celiac Disease Autoantigen by Transient Expression in *Nicotiana benthamiana*

**DOI:** 10.3389/fpls.2015.01067

**Published:** 2015-12-01

**Authors:** Vanesa S. Marín Viegas, Gonzalo R. Acevedo, Mariela P. Bayardo, Fernando G. Chirdo, Silvana Petruccelli

**Affiliations:** ^1^Centro de Investigación y Desarrollo en Criotecnología de Alimentos (CIDCA), Consejo Nacional de Investigaciones Científicas y Técnicas (CONICET) – Departamento de Ciencias Biológicas, Facultad de Ciencias Exactas, Universidad Nacional de La Plata (UNLP)La Plata, Argentina; ^2^Instituto de Investigaciones en Ingeniería Genética y Biología Molecular (INGEBI), Consejo Nacional de Investigaciones Científicas y TécnicasBuenos Aires, Argentina; ^3^Instituto de Estudios Inmunológicos y Fisiopatológicos (IIFP), Consejo Nacional de Investigaciones Científicas y Técnicas – Departamento de Ciencias Biológicas, Facultad de Ciencias Exactas, Universidad Nacional de La PlataLa Plata, Argentina

**Keywords:** human tissue transglutaminase, celiac disease, secretory pathway, vacuolar sorting, elastin-like polymer

## Abstract

Celiac Disease (CD) is a gluten sensitive enteropathy that remains widely undiagnosed and implementation of massive screening tests is needed to reduce the long term complications associated to untreated CD. The main CD autoantigen, human tissue transglutaminase (TG2), is a challenge for the different expression systems available since its cross-linking activity affects cellular processes. Plant-based transient expression systems can be an alternative for the production of this protein. In this work, a transient expression system for the production of human TG2 in *Nicotiana benthamiana* leaves was optimized and reactivity of plant-produced TG2 in CD screening test was evaluated. First, a subcellular targeting strategy was tested. Cytosolic, secretory, endoplasmic reticulum (C-terminal SEKDEL fusion) and vacuolar (C-terminal KISIA fusion) TG2 versions were transiently expressed in leaves and recombinant protein yields were measured. ER-TG2 and vac-TG2 levels were 9- to 16-fold higher than their cytosolic and secretory counterparts. As second strategy, TG2 variants were co-expressed with a hydrophobic elastin-like polymer (ELP) construct encoding for 36 repeats of the pentapeptide VPGXG in which the guest residue X were V and F in ratio 8:1. Protein bodies (PB) were induced by the ELP, with a consequent two-fold-increase in accumulation of both ER-TG2 and vac-TG2. Subsequently, ER-TG2 and vac-TG2 were produced and purified using immobilized metal ion affinity chromatography. Plant purified ER-TG2 and vac-TG2 were recognized by three anti-TG2 monoclonal antibodies that bind different epitopes proving that plant-produced antigen has immunochemical characteristics similar to those of human TG2. Lastly, an ELISA was performed with sera of CD patients and healthy controls. Both vac-TG2 and ER-TG2 were positively recognized by IgA of CD patients while they were not recognized by serum from non-celiac controls. These results confirmed the usefulness of plant-produced TG2 to develop screening assays. In conclusion, the combination of subcellular sorting strategy with co-expression with a PB inducing construct was sufficient to increase TG2 protein yields. This type of approach could be extended to other problematic proteins, highlighting the advantages of plant based production platforms.

## Introduction

Celiac disease is a chronic disorder caused by the ingestion of prolamins from wheat, barley, rye, or oats, which affects around 1% of the general population (Abadie et al., 2011). Although CD presents extremely heterogeneous clinical spectrum, it can be estimated that only 1 out of 7 patients are actually diagnosed (Rubio-Tapia et al., 2009), therefore massive serological screenings could increase the detection of CD and improve celiac patients’ life quality. Among diagnostic serological tests, detection of anti-TG2 IgA has the highest sensitivity and specificity therefore is the single most efficient serological test to screen in risk populations (Husby and Murray, 2014). Cost considerations are important for the implementation of widespread screening programs (Viljamaa et al., 2005), for that reason availability of a low cost and high quality source of TG2 antigen for massive CD serological screening assays is attractive.

Different human TG2 production systems have been assayed such as *Escherichia coli* (Shi et al., 2002), insect cells (Osman et al., 2002), human embryonic kidney cells (Sardy et al., 1999), and plant cells (Sorrentino et al., 2005, 2009). Low recombinant protein yields are generally obtained since TG 2 cross-linking activity has toxic effects on cell growth and development (Griffin et al., 2002). In tobacco Bright Yellow 2 (BY-2) cells, TG2 accumulation was higher when the protein was targeted to the apoplast (apo) than when it was fused to a chloroplast (chl) sorting signal and partial degradation of both apo-TG2 and chl-TG2 was detected (Sorrentino et al., 2005). No transgenic BY-2 clones were obtained for the cytosolic TG2 construct probably due to toxic effect of this enzyme, which might prevent regeneration and growth of the transformed BY-2 cells (Sorrentino et al., 2005). In transgenic tobacco plants, apo-TG2 accumulated at higher levels than the one sorted to the cytosol and chl compartments (Sorrentino et al., 2009). Although plant-produced TG2 was recognized by IgA serum of celiac patients (Sorrentino et al., 2005) no further efforts to produce TG2 in plants were reported. Plants are a cost effective platform for the production high-value recombinant proteins for industrial and clinical applications (Gleba and Giritch, 2014; Makhzoum et al., 2014). Several plant-produced recombinant proteins are commercially available including glucocerebrosidase (the first plant-made biologic approved by the US Food and Drug Administration), veterinary pharmaceuticals, technical enzymes, research reagents, media ingredients, and cosmetic products (Sack et al., 2015). Plant-based platforms are diverse in terms of plant species, cell or organs used for the production and technology used to achieve the over-expression of the gene of interest (Sack et al., 2015). Numerous factors have a profound impact in protein accumulation levels among then protein stability (Egelkrout et al., 2012). Stability can be increased using different subcellular targeting strategies such as accumulating proteins in the apo, ER, vacuoles, chl, on the surface of oil bodies, as well as expression in different organs such as seeds, leaves, and hairy roots (Hood et al., 2014) or fusion to insoluble tags such as ELPs, hydrophobins, and zeins (Floss et al., 2010; Joensuu et al., 2010). Within the endomembrane system, several strategies improve foreign protein accumulation such as ER retention (Wandelt et al., 1992; Fiedler et al., 1997), vacuolar sorting (Stoger et al., 2005; Shaaltiel et al., 2007) and inhibition of apoplast protease activity (Benchabane et al., 2008; Goulet et al., 2012), The results obtained using the subcellular targeting strategies or fusion to insoluble tags depend on the nature of the heterologous protein. These stabilizing strategies have yet not been tested to increase TG2 accumulation in plants.

In this work two strategies were evaluated to increase accumulation TG2 in tobacco leaves. The first one was compartmentalization of TG2 inside the secretory pathway to avoid its cytosolic toxicity and also apoplast degradation. TG2 was fused to a SEKDEL ER retention sequence and also KISIA CT terminal VSS (Petruccelli et al., 2007). The second strategy was the induction of protein body formation in the ER by co-expression of TG2 with a novel, highly hydrophobic elastin-like polymer. TG2 expressed using these strategies was purified and its performance as antigen in serological assays was evaluated.

## Materials and Methods

### Antibodies

Three anti-TG2 monoclonal antibodies (mAb) named 2G3, 5G7, and 4E1 were produced by Dr. F Chirdo. The mAb recognizes different epitopes: 2G3 (aa 314–329), 5G7 (aa 548–558), and 4E1 (aa 637–648) (Di Niro et al., 2005). Human serum samples were obtained using the conventional procedure for CD diagnosis. Patients signed a written consent. The study was approved by the Ethical Committees of the Hospital Interzonal General de Agudos (HIGA), General San Martin de La Plata, Buenos Aires, Argentina. Celiac patients were diagnosed on the basis of the clinical findings, histological examination, and positive serology. Negative control sera were taken from healthy non-celiac volunteers. CD patients and controls serum samples belongs to sera bank characterized at Instituto de Estudios Inmunológicos y Fisiopatológicos, (IIFP). Other antibodies utilized for this study were mouse anti-GFP antibody (# G1546, Sigma–Aldrich, St. Louis, MO, USA), rabbit anti-RFP antibody (# R10367, Thermo Scientific Pierce, Rockford, IL, USA), goat anti-mouse IgG (H+L) secondary antibody biotin conjugate (# 31802, Thermo Scientific Pierce, Rockford, IL, USA), goat anti-rabbit IgG (H+L) secondary antibody biotin conjugate (# 31820, Thermo Scientific Pierce, Rockford, IL, USA), high sensitivity streptavidin HRP Conjugate (# 21130, Thermo Scientific Pierce, Rockford, IL, USA) and mouse anti-human IgA secondary antibody HRP conjugate (#SA135467, Thermo Scientific Pierce, Rockford, IL, USA).

### Plants

*Nicotiana benthamiana* plants were grown in a growth chamber at 22°C 16-h-light/8-h-dark cycles. Six to eight week-old plants were used for each set of experiments and infiltrations were performed in the third and the fourth leaves counting top down starting with the youngest mature leaf.

### TG2 Constructs

The cDNA encoding TG2 (GenBank Accession Number GI 50593093) from Caco 2 (Human colonic carcinoma) cell line (Bayardo et al., 2012) was amplified with the oligonucleotide primers forward F-SP-TG2 (GTGGGTACCCAATGGCCGAGGAGCTGGTC) and reverse R-TG2HisSal (CCCGTCGACGTGGTGGTGGTGGTGGTGGGCGGGGCCAATGATGAC), designed to place TG2 in frame with the sequence encoding a mouse immunoglobulin heavy chain signal peptide (SP; MGWSWIFLFLLSGAAGGY) from pRTL202 (Restrepo et al., 1990) and to introduce the sequence encoding a six histidine purification tag at the TG2 sequence 3′ end. The PCR product was digested with *Kpn* I and *Sal* I and cloned into pRTL-G-KDEL and pRTL-G-KISIA (Petruccelli et al., 2007) to produce p-ER-TG2 and p-vac-TG2, respectively. To generate a secretory version TG2 was amplified with F-SP-TG2 and R-HISSTOP (CCCGTCGACTCAGTGGTGGTGGTGGTGGTGGGC) and cloned into p-secG (Petruccelli et al., 2007). The cassettes CaMV35S promoter::SP-TG2-His (STOP or KDEL)::Nos transcription terminator signal were released from these vectors by digestion with *Hind* III and *Sal* I and subcloned into the binary vector pBLTi-121 (Pagny et al., 2000). To produce the cytosolic version, the SP encoding sequence was removed by releasing TG2 from p-sec-TG2-His with *Kpn* I and *Sal* I and subcloning it into pBLTi 121, digested with the same enzymes.

The vacuolar version of TG2 was amplified with the primers F-SP (CACCATGGGCTGGAGCTGGATC) and R-Ter (CTAGGCGGGGCCAATGATGAC) and the PCR product was directionally cloned into pENTR/D TOPO (Life Technologies, S.A. Buenos Aires, Argentina) to introduce attL1 and attL2 recombination sites and finally was transferred to the binary destination vector pGWB2 (Nakagawa et al., 2007) using LR site specific clonases. To fuse ER-TG2 to a fluorescent protein, the sequence encoding mCherry from ER-Cherry (Nelson et al., 2007) was amplified with F-SP-Cherry (CACCCTCGAGCCGACCTCGACCTAGAAAGAGAAGGAGGACAGTCCTTCGACGTCCATGGTGAGCAAGGGCGAGGAG) and R-Cherry (TATTAAGCTTGGTACCCAGGTGGACCTGGAGGCCATGCCGCCGGTGGAGTG) and the PCR product was cloned into pENTR/D TOPO. Then the ER-TG2 sequence was released from p-TG2 with *Kpn* I and *Hind* III and introduced into pENTR-SP-RFP, cutted with the same enzymes. Finally the LR recombination reaction was performed between pENTR-SP-ER-RFP-TG2 and pGWB2 (Nakagawa et al., 2007) to obtain ER-RFP-TG2 in a binary vector.

### Elastin-like Polymer Constructs

A novel synthetic ELP gene encoding for 36 repeats of the pentapeptide VPGXG in which the guest residue X were V and F in ratio 8:1 [V8F1] were purchased to GenScript Corp (Piscataway, USA). This synthetic ELP gene was designed to be expressed into the plant secretory pathway by introduction of the sequences encoding for a mouse immunoglobulin G1 SP (Petruccelli et al., 2006) and the ER retention sequence SEKDEL, upstream and downstream, respectively (Supplementary Figure S1). To facilitate purification the sequence encoding a hexahistidine tag was introduced between the ones encoding ELP[V8F1] and SEKDEL. To allow further multimerization the *Pfl* MI and *Bgl* I restriction sites where incorporate at the beginning and end of the ELP[V8F1] encoding sequence. Codon use was optimized for *Nicotiana benthamiana*^1^. Potential splices sites and inverted repeats were reduced and GC content was adjusted by a GenScript in-house algorithm. ELP constructs was introduced into the plant binary expression vector pEAQ- HT-DEST1 (Sainsbury et al., 2009) using LR site specific clonases (Life Technologies, S.A. Buenos Aires, Argentina).

### *Agrobacterium* Infiltration

*Agrobacterium tumefaciens* strain GV3101 harboring the pGWB2-sec-TG2, pGWB2-cyto-TG2, pGWB2- ER-TG2, pGWB2-vac-TG2, pGWB2-ER-RFP-TG2, or Tomato Bushy Stunt Virus P19 (Voinnet et al., 2003) binary plasmids were grown in YEB media (5 g/L beef-extract, 1 g/L yeast-extract, 5 g/L peptone, 5 g/L sucrose, 2 mM MgSO_4_) at 28°C overnight. Cells where then centrifuged at 5,000 × *g* and resuspended in IM [10 mM MgCl_2_, 10 mM 2-(*N*-morpholino)ethanesulfonic acid (MES) pH 5.7, 200 μM acetosyringone] adjusting agrobacterium OD_600_ to 0.3 for TG2 constructs, 0.2 for pEAQ1-ELP, 0.1 for ER-GFP (Haseloff et al., 1997), sec-RFP (Scabone et al., 2011), and P19. The bacterial suspensions were incubated at least three hours at 28°C prior to infiltration. Leaf infiltration was performed manually using disposable, needleless 1 mL syringes with which pressure was applied between ribs at the abaxial face of the leaf.

### Enzyme-linked Immunosorbent Assay (ELISA)

*Nicotiana benthamiana* leaf samples were collected at 5 d.p.i., since maximum TG2 levels were detected at this time in expression kinetics experiment. At least three biological replicates per sample were performed. Each replicate contained five leaf pieces of the infiltrated tissue from different plants. Each sample was analyzed by Enzyme-linked Immunosorbent Assay (ELISA) in triplicate. Leaves were frozen with liquid nitrogen and grounded. The powder was suspended in extraction buffer (20 mM sodium phosphate, 0.5 M sodium chloride pH 7.5) for 15 min at 4°C. After centrifugation at 10,000 × *g* protein concentration in the supernatant was measured by Bradford assay (Bradford, 1976) using bovine serum albumin as standard. Plastic wells were coated with the same amount of total leaf extract (∼100 μg TSP) or 1 μg of leaf purified TG2 in PBS with 5 mM CaCl_2_ by passive adsorption at 4°C overnight (Sulkanen et al., 1998). Plates were then blocked with 5% non-fat milk solution for 1 h at 37°C. Then 100 μL of a 1:500 dilution of TG2 mAb 2G3 (Di Niro et al., 2005) or a 1:50 diluted pool of 12 patient sera were added and incubated for 16 h at 4°C as primary antibody. Then biotin-conjugated anti-mouse IgG and HRP-conjugated streptavidin or HRP-conjugated anti-human IgA was applied for 1 h at 37°C as the secondary antibody. Color was developed with tetramethylbenzidine (TMB)–peroxidase substrate (Kirkegaard and Perry Laboratories, Gaithersburg, MD, USA) and the optical density was measured at 630 nm wavelenght.

### Western Blotting

Total soluble proteins were extracted from *N. benthamiana* agroinfiltrated leaves by grinding 500 mg of leaves in 0.5 mL SDS PAGE sample buffer (72 mM Tris-HCl, 2% SDS, 10% glycerol, 5% β-mercaptoethanol, pH 6.8). The crude extract was boiled for 5 min and centrifuged (20 min, 13,000 rpm, RT). Supernatant samples (20 μL) were separated by electrophoresis on a 10% polyacrylamide gel. For quantitative analysis, the amount of crude extract was adjusted to load the same amount of RLS. After gel electrophoresis, proteins were transferred to nitrocellulose membranes. Blocked membranes (5% non-fat milk solution) were incubated with 1:500 dilution of anti-TG2 mAb 2G3, 5G7, or 4E1 (Di Niro et al., 2005) overnight at 4°C, followed by incubation with a biotinylated goat anti-mouse IgG antibody (1:20,000), 1 h at 37°C, and with HRP-conjugated streptavidin (1:20,000) 30 min at 37°C. Finally, chemiluminescence was generated by addition of 1.25 mM luminol (#A8511 Sigma–Aldrich, St. Louis, MO, USA), 200 μM *p*-coumaric acid (#C9008, Sigma–Aldrich, St. Louis, MO, USA), 0.09% [v/v] H_2_O_2_, 0072% [v/v] DMSO, 100 mM Tris-HCl pH 8.5 substrate, and luminescent signal was captured using X-ray film (Amersham Hyperfilm ECL, GE Healthcare Life Sciences, Argentina). The film was scanned and protein band intensity was measured using ImageJ software^2^.

### TG2 Purification

Transglutaminase 2 was purified from leaves co-infiltrated with *Agrobacterium* suspensions carrying pGWB2-ER-TG2 or pGWB2- vac-TG2 and pEAQ1-ELP. Tobacco leaves (20 g) were frozen with liquid nitrogen and grounded into a fine powder using mortar and pestle. The powdered tissue was extracted with 20 mL of extraction buffer for 15 min at 4°C. After centrifugation at 10,000 × *g* the supernatant was incubated for 1 h at 4°C with 50 μL Ni Sepharose (GE Healthcare Life Sciences, Argentina) and proteins bound to Ni Sepharose were retained using Micro Bio-Spin columns (Bio-Rad, Hercules, CA, USA), washed three times with extraction buffer and TG2 was eluted with 0.2 M NaH_2_PO_4_ pH 4.5 and neutralized with NaHCO_3_. Protease inhibitor cocktail (Roche Applied Science, Mannheim, Germany) was added to the eluted TG2 fraction. Protein concentration was measured using a NanoDrop 2000 UV/Visible Spectrophotometer (Thermo Scientific, Rockford, IL, USA) and purity was analyzed by SDS-PAGE.

### Protein Quantification

Endoplasmic reticulum-green fluorescent protein and sec-RFP quantification was performed by fluorometry of leaf extracts with Synergy microplate reader (Biotek Instruments, Inc., Winooski, VT, USA). Two hundred μL/well of each extract were added to a 96 well black plate. GFP fluorescence was measured by using excitation at 485 nm and emission at 516 nm, and RFP fluorescence by using excitation at 563 nm and emission at 610 nm. Arbitrarily, 1 unit of fluorescence was assigned to each of the samples obtained from leaf in the absence of ELP, and then both with or without ELP samples were normalized to these extracts. TG2 quantification was performed by a calibration curve obtained with the plant purified TG2. To this end, different amounts of purified TG2 were loaded into the gel to make the calibration curve and 20 μL of total leaf extracts to be quantified were loaded on the same gel. After transfer, an immunoblot was performed as describe above. The signal obtained for the 20 μL of total leaf extracts were transformed into ng TG2 using the obtained calibration curve.

### Confocal Analysis and Image Processing

Abaxial epidermal cells of agroinfiltrated leaves were observed between 3 and 7 d.p.i. with a Confocal Laser Scanning Microscope (CLSM) LEICA TCS SP5 AOBS (Advanced Microscopy Facility, FCE, UNLP), using a 63× oil immersion objective. GFP was excited at 488 nm (Argon 100 mW Laser) and detected in the 496–532 nm range. RFP was excited at 543 nm (HeNe 1.5 mW Laser) and detected in the 570–630 nm range. Simultaneous detection of GFP and RFP was performed by combining the settings indicated above in a sequential scanning set-up, as instructed by the manufacturer. All images shown were acquired using the same photomultiplier gain and offset settings. Post-acquisition image processing was performed with ImageJ software^2^.

### Statistical Analysis

All statistical analyses were carried out using Prism 6 (GraphPad Software, GraphPad Inc., La Jolla, CA, USA). One-way ANOVA test and Tukey’s multiple comparisons test were used to determine means with statistical differences. Alternative Student’s *t*-test was performed. A *p*-value < 0.05 was regarded as statistically significant.

## Results

### Accumulation of TG2 Fused to Different Sorting Signals

**FIGURE 1:**
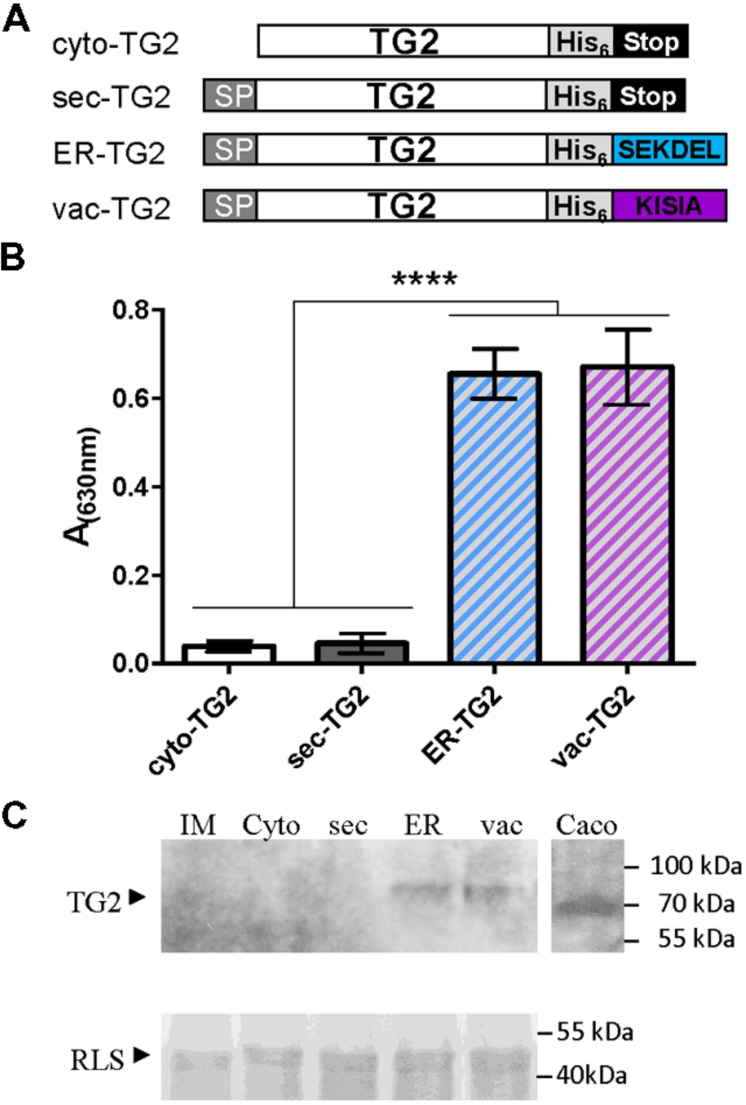
**Subcellular targeting strategies tested for stabilize TG2 in leaves. (A)** Schematic representation of the TG2 constructs used for *Agrobacterium*-mediated transient expression in *Nicotiana benthamiana* leaves. Cyto-TG2 is a cytosolic form of TG2. Sec-TG2, ER-TG2, and Vac-TG2 are introduced in the secretory pathway with murine signal peptide (SP) from gamma 1 antibody chain; SEKDEL, ER retention SP; KISIA is a CT vacuolar targeting signal of the amaranth 11S globulin. Scheme is not drawn to scale. **(B)** Enzyme-linked Immunosorbent Assay (ELISA) of TG2 fused to the different sorting signals. Microwells were coated with the same amount of total leaves extract overnight at 4°C. After blocking, anti-TG2 mAb 2G3 was added, followed of incubation with a biotin-conjugated anti-mouse, later with HRP-conjugated streptavidin and developed with TMB peroxidase substrate. Three biological replicates (each replicate containing five leaf disks of the infiltrated tissue from a different plant) were used for ELISA. Error bars represent the standard error of the mean (SEM). ^∗∗∗∗^Denotes statistically significant difference by Tukey’s multiple comparisons test (*P* < 0.001). **(C)** Western blot of TG2 fused to the different sorting signals. Expression levels were measured by scanning densitometry of Western Blot developed with 2G3 mAb with a minimum of three independent experiments. The amount of total extract was adjusted using RLS stained with Coomassie Brilliant Blue R-250 as loading control.

Although TG2 from Caco2 cell line was cloned in *E. coli* expression vector and different conditions were assayed to produce it, low recovery levels were obtained. For that reason in this work, we attempted to produce TG2 in plant cells. To this end, four versions of TG2 in plant expression binary vector were obtained: cytosolic (cyto-TG2), secretory (sec-TG2), ER-TG2, and vacuolar (vac-TG2), and their schematic representations are shown in **Figure 1A**. For vacuolar sorting the C terminal KISIA sequence from the amaranth 11S storage globulin was added to TG2 (Petruccelli et al., 2007). To facilitate purification a six histidine tag was also fused to TG2 (**Figure 1A**). The four TG2 constructs were introduced in *A. tumefaciens* GV3101 and leaves of *N. benthamiana* were infiltrated with these agrobacteria. Five days after infiltration, leaves were collected and accumulation of TG2 was measure by ELISA using anti-TG2 mAb 2G3. **Figure 1B** shows that the highest accumulation level was obtained for ER-TG2 and vac-TG2, and that cyto-TG2 and sec-TG2 levels were approximately 9- to 16-fold lower than those of the ER and vac variants. No significant differences were observed between ER-TG2 and vac-TG2, suggesting that fusion to either SEKDEL or KISIA C-terminal signals is equally efficient to increase TG2 accumulation levels. A kinetic analysis of ER-TG2 and vac-TG2 expression showed that maximum accumulation was reached at 5 d.p.i (Supplementary Figure S2).

Total leaf extracts were also analyzed by Western Blot using mAb 2G3 as detection antibody (**Figure 1C**). Caco2 total extract was also loaded into the gel as positive control. Cyto-TG2 and sec-TG2 were not detected while ER-TG2 (81,4 kDa) and vac-TG2 (81,2 kDa) variants had the expected size suggesting that both forms accumulated in leaves in a stable way. The amount of proteins quantified by immunoblot followed by densitometry analysis showed not significant differences in the accumulation levels of ER-TG2 and vac-TG2 in good correlation with ELISA test.

### Effect of a Novel Hydrophobic ELP on the Accumulation of TG2

**FIGURE 2:**
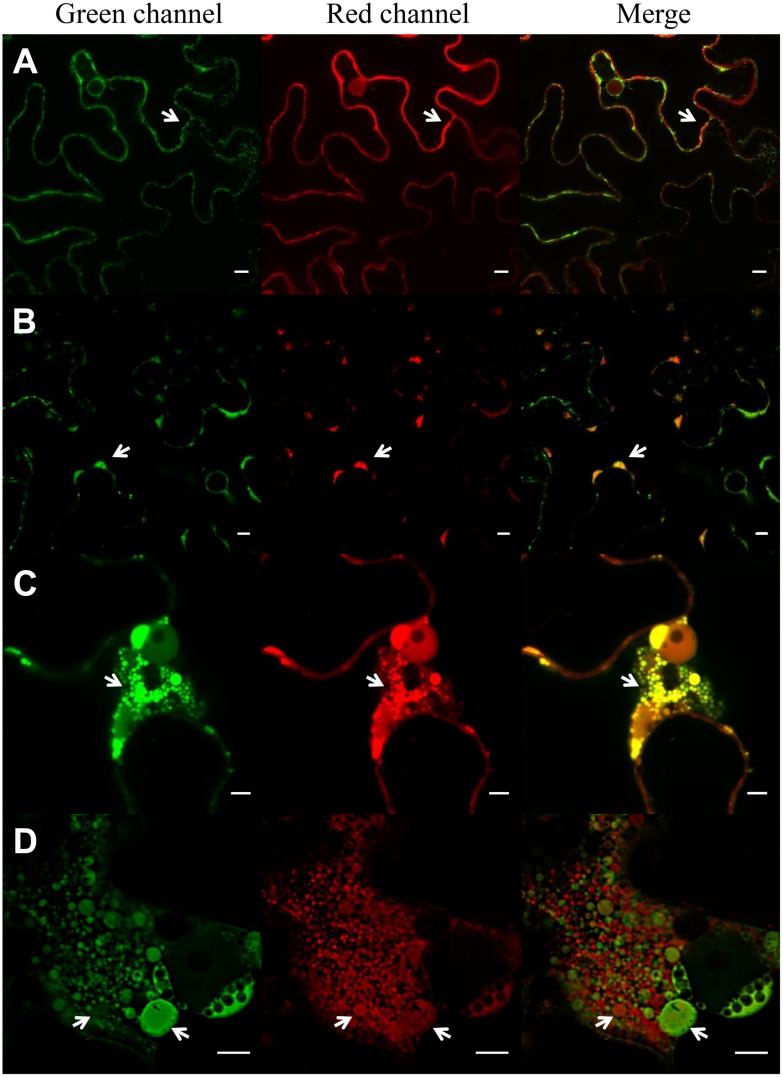
**Induction of protein bodies by expression of ELP.** Expression of ER-GFP and sec-RFP **(A)**, ER-RFP-TG2 and ER-GFP **(B)**, ER-GFP, sec-RFP and ELP **(C)**, ER-RFP-TG2, ER-GFP and ELP (D) in *N. benthamiana* leaf epidermal cells. ER-GFP shows the typical ER reticulated pattern (green channel, **A**), sec-RFP has an irregular pattern on the borders of the cell typical of apoplast (apo; red channel, **A**), no co-localization of ER-GFP and sec-RFP is observed in the merge panel. ER-RFP-TG2 **(B)** is mainly located in clusters (PB) in the borders of the cells (arrows), and the signal on the rest of the ER network is low. Co-localization of ER-RFP-TG2 and ER-GFP is observed mainly in these clusters (**B**, merge). ELP expression induces protein body formation (PB) in **(C,D**). Co-localization of ER-GFP and sec-RFP is observed in PB (**C**, merge) while RFP-TG2 did not entirely colocalize with ER-GFP (**D**, merge) in the nuclear region of the cell. Scale bars: 10 μm **(A,B)** and 5 μm **(C,D)**.

A novel ELP construct consisting in 36 repeats of the pentapeptide VPGXG in which the guest residues X were V and F in ratio 8:1 (Supplementary Figure S1) with a theoretical inverse phase transition temperature (T_t_) of 18°C (Urry et al., 1992) and which is expected to be insoluble at *N. benthamiana* growing conditions was used in this work. The ELP was sorted to the ER by means of a secretory SP and SEKDEL ER retention sequence. The ability of this ELP to induce protein body formation was analyzed with CLSM, using GFP-HDEL (Haseloff et al., 1997) and sec-RFP (Scabone et al., 2011) as fluorescent markers of the secretory pathway. **Figure 2A** shows that ER-GFP had a normal reticular pattern in the absence of ELP and that sec-RFP localized on the borders of the cell with an irregular pattern typical of apoplast accumulation. ER-RFP-TG2 had also a reticular pattern but its accumulation produced clusters on the borders of the cells (**Figure 2B**, arrows). A partial co-localization was observed between ER-GFP and ER-RFP-TG2 in the merge panel, ER-RFP-TG2 was located mainly in the clusters while ER-GFP had an uniform distribution (**Figure 2B**). Accumulation of ER-RFP-TG2 fusion was approximately 8,4 ± 1,8 μg/g fresh leaf tissue. When ER-GFP and sec-RFP were co-expressed with ELP, large ER-PB were observed predominantly close to the nuclei and in cortical regions (**Figure 2C**). A co-localization pattern of sec-RFP in transit with ER-GFP was found as can be observed in yellow in the merge panel (**Figure 2C**). Nevertheless ELP did not affect final localization of sec-RFP since the apo pattern was also observed for this construct (Supplementary Figure S3). Some of the ELP induced PBs were larger than the nucleolus (**Figure 2C**). When ER-RFP-TG2 was co-expressed with ELP, small (less than 1 μm) and large PBs were also observed (**Figure 2D**) but only a partial co-localization with ER-GFP was detected (**Figure 2D**, merge panel). PBs, in the nuclear region, had heterogeneous size and composition distribution since some of them had only ER-RFP-TG2 and other only ER-GFP. In contrast, in the cortical region, a complete co-localization of green ER-GFP PBs and red ER-RFP-TG2 PBs was observed (Supplementary Figure S4). The integrity of ER-RFP-TG2 was confirmed by immunoblot analysis to ensure that the red fluorescence corresponded to entire fusion protein (Supplementary Figure S5).

**FIGURE 3:**
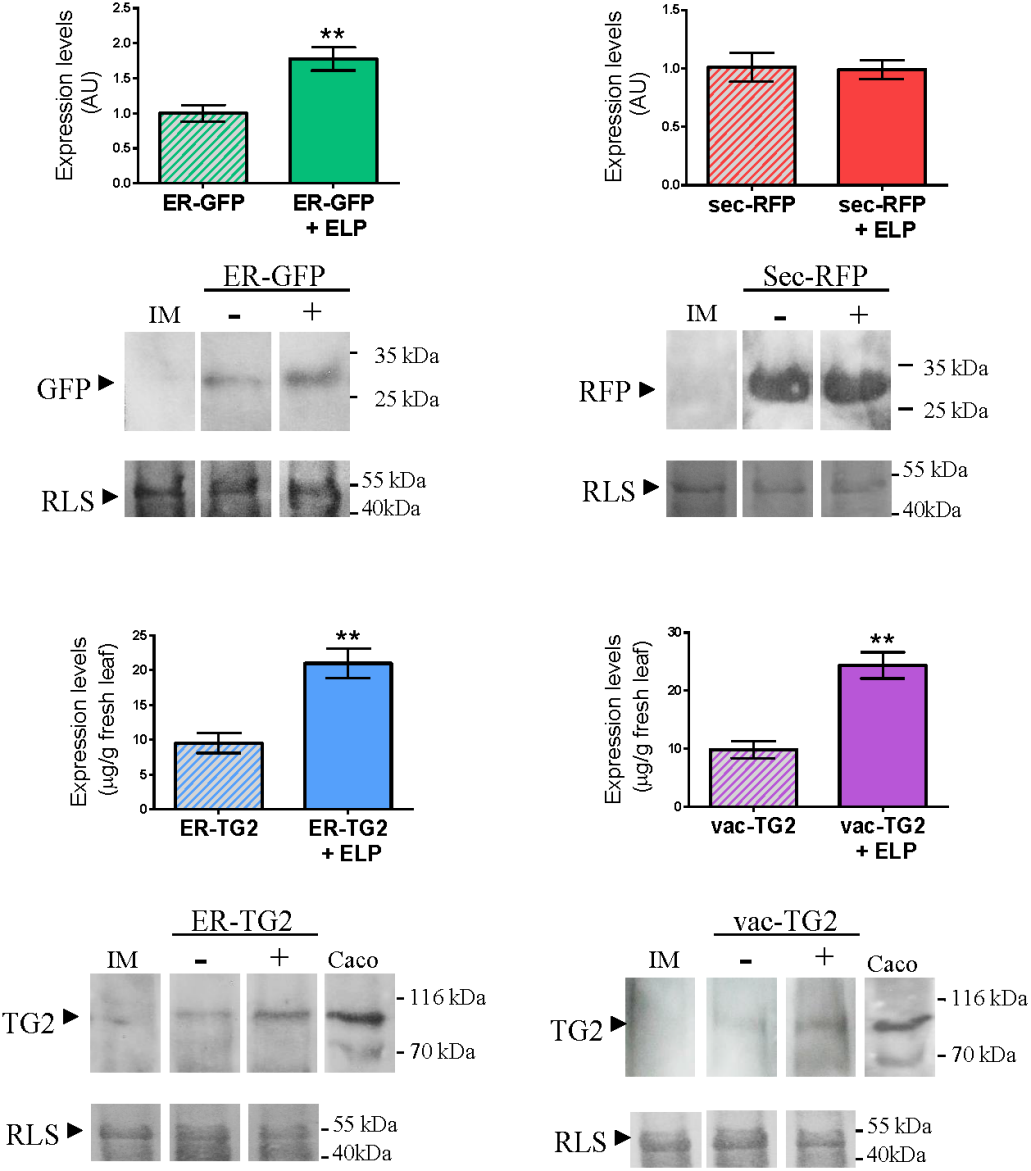
**Effect of ELP on the accumulation levels of ER-GFP, sec-RFP and ER-TG2 and vac-TG2.** The same amount of total extract was loaded on the gel as can be observed by the amount of RLS stained with Coomassie Brilliant Blue R-250. The immunoblot was developed with anti-GFP, anti-RFP, and anti-TG2 mAb 2G3, for ER-GFP, sec-RFP and ER-TG2, and vac-TG2, respectively. The intensity of the band was quantified by using ImageJ. Three biological independent experiments were performed and each replicate was obtained using leaves from five different plants. Error bars are standard error of the mean (SEM). ^∗∗^Denotes statistically significant difference by Student’s *t*-test (*P* < 0.01).

To evaluate the impact of ELP on the accumulation of ER-GFP, sec-RFP, ER-TG2, and vac-TG2 a Western Blot was performed. The same amount of total leaf extracts was load into the gel and as control the amount of RLS in each lane is shown. The intensity of the GFP, RFP, and TG2 bands were quantify as is detailed in Section “Materials and Methods” and the obtained results are shown in **Figure 3**. For ER-GFP a 2.0-fold increase in the accumulation level was observed by ELP induced PB formation, while not significant differences were found in sec-RFP levels (**Figure 3**, upper panel). Accumulation levels of ER-TG2 and vac-TG2 were modified from 9,5 ± 1,5 and 9,9 ± 1,4 to 20,9 ± 2,1 and 24,4 ± 2,3 μg/g fresh leaf tissue, respectively, by expression of ELP (**Figure 3**, lower panel). In conclusion, ELP induced 2.1- and 2.5-fold increase in the accumulation of ER-TG2 and vac-TG2, respectively.

### ER-TG2 and vac-TG2 as Antigen for CD Diagnosis

**FIGURE 4:**
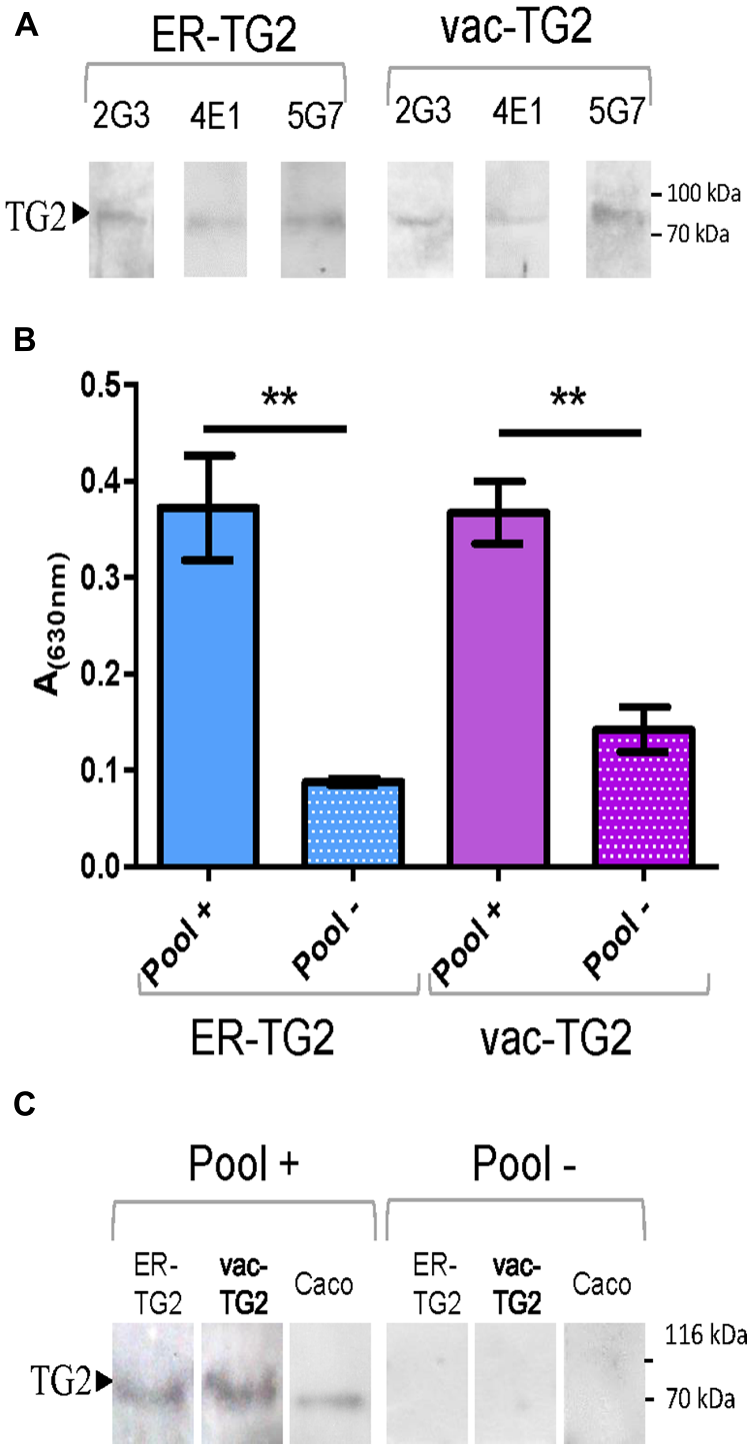
**Plant purified ER-TG2 and vac-TG2 as antigen for EC diagnosis. (A)** Recognition of ER-TG2 and vac-TG2 by three different monoclonal antibodies (mAb). **(B)** ELISA with IgA sera of celiac patient and normal healthy donors. ^∗∗^Denotes statistically significant difference by Student’s *t*-test (*P* < 0.01). **(C)** Immunoblot with a pool of IgA sera of celiac patient (Pool+) and normal healthy donors (Pool–). The protein loaded on the gel: ER-TG2, vac-TG2, caco extract is indicated at the top of the immunoblot.

Endoplasmic reticulum-transglutaminase 2 and vac-TG2 were purified from leaves using immobilized metal ion affinity chromatography. To test their usefulness as antigen their recognition by the mAbs 2G3, 5G7, or 4E1, which recognize different TG2 epitopes, was analyzed by immunoblot. Both vac-TG2 and ER-TG2 were positively recognized by these antibodies as is shown **Figure 4A**, confirming that although in humans TG2 is a localized in the cytosol, the introduction into the plant secretory pathway do not affect the structure of the epitopes recognized by these mAbs. In order to test the performance of the plant purified ER-TG2 and vac-TG2 version in CD screening test an ELISA was performed using a pool of 12 sera of CD patient and control healthy donors (**Figure 4B**). We found that the pool of CD sera recognized both ER-TG2 and vac-TG2 with a large significant difference over the value obtained for the healthy donors. Plant purified TG2 recognition was also assayed by Western Blot (**Figure 4C**) confirming that the full-length ER- and vac-TG2 variants were recognized by CD sera while not recognition occurred for control sera. Therefore the plant-produced ER- and vac-TG2 versions conserved the epitopes recognized by IgA sera of celiac individuals. These results point out the usefulness of plant-produced TG2 for develop CD screening tests.

## Discussion

In this work we showed that TG2 fused to the C terminal KDEL or KISIA sorting signals accumulated at significantly higher levels than the cytosolic and apoplast versions, confirming the convenience of testing different subcellular compartments as strategy to increase accumulation levels. In leaves, the ER is a favorable destination for many proteins such as vicilin, single chain, and full length antibodies, truncated version influenza hemagglutinin (Wandelt et al., 1992; Schouten et al., 1996; Fiedler et al., 1997; Petruccelli et al., 2006; Mortimer et al., 2012) and although fusion to KDEL/HDEL signals not always enhance recombinant protein accumulation, it is frequently used for subcellular targeting strategies (Boothe et al., 2010; Hood et al., 2014). In contrast with ER retention, sorting of foreign proteins to leaf plant central vacuole has been less studied as sorting strategy. The plant vacuole is one of the largest subcellular compartments that storage ions and metabolites (Marty, 1999). Although it is considered a hostile environment for foreign protein accumulation (Hood et al., 2014), some proteins accumulate at high levels in central vacuoles such as glucocerebrosidase in carrot cells (Shaaltiel et al., 2007), IgG in tobacco BY2 cells (Misaki et al., 2011), human alpha-mannosidase in tobacco leaves (De Marchis et al., 2013), human complement factor C5a in both *N. tabacum* and *N. benthamiana* leaves (Nausch et al., 2012) and human collagen in tobacco leaves (Stein et al., 2009). Other proteins such as human IgG1 and G4 have higher apo yield compared to the accumulation in ER and vacuoles in carrot suspension cell cultures (Shaaltiel et al., 2006). For synthetic spider silk ER-targeted variant was more abundant than the vacuolar variant in *Arabidopsis* leaves (Yang et al., 2005). In opposition to leaf tissues or suspension cultures, there are numerous examples of foreign proteins that stably accumulate in storage vacuoles in seeds (Stoger et al., 2005; Khan et al., 2012).

Although the nature of VSS employed to target foreign proteins to vacuoles might have an impact on protein stability, there are examples of enhanced accumulation for heterologous proteins fused to different types of VSSs. For example stable deposition was obtained for proteins fused to different CT-VSSs such as tobacco chitinase A CT (DLLVDTM) for glucocerebrosidase (Shaaltiel et al., 2007), phaseolin CT (AFVY) for human complement factor C5a (Nausch et al., 2012), and amaranth 11S globulin CT (KISIA) in this work. Furthermore, ssVSSs had also a positive impact on the accumulation of foreign proteins such as aleurain ssVSS (NPIR) and sporamin ssVSS (NPIRL), which improved build-up of human collagen (Stein et al., 2009) and IgG (Misaki et al., 2011), respectively. Increased vacuolar accumulation was also observed for human alpha-mannosidase, whose N-terminal sequence sorted it directly to the vacuole bypassing the Golgi apparatus (De Marchis et al., 2013). It is believed that VSSs are necessary for post-Golgi sorting to vacuoles and that the traffic pathway can affect foreign protein stability since the pH varies along the secretory pathway (Neuhaus and Martinoia, 2011; Shen et al., 2013). The different data published for vacuolar sorted foreign proteins, indicate that reaching a stable accumulation in vacuoles is more dependent on the nature of the foreign protein than the sorting signal used.

In leaves, several fusion tags such as ELP, hydrophobins (HFBI), and N terminal proline-rich region of gamma zein (Zera) improve accumulation levels of recombinant fusion partners (Conley et al., 2011). These three fusion tags are supposed to increase accumulation of recombinant proteins by inducing the formation of leaf PB, that are similar to prolamin PB found in seeds, where recombinant proteins are protected from proteolytic degradation (Conley et al., 2009). Recently, it has been reported that PB formation is not exclusively promoted by the fusion tags and that protein accumulation level is a critical factor to trigger PB formation (Saberianfar et al., 2015). Both ER-GFP and fungal xylanases unfused to these tags are able to induce PB formation when their accumulation levels were higher than 0.2% of TSP (Saberianfar et al., 2015). In this work, we showed that ER-RFP-TG2 induced PB formation on the cortical region of the leaf epidermal cells although protein accumulation level was lower than 0.2% of TSP. Even though protein accumulation level is an important aspect for PB formation, other characteristics of the heterologous protein such as aggregation tendency or recruitment of foldases and chaperone might be also involved in this phenomenon.

A novel highly hydrophobic ELP (VPGXG)_36_ [where X = V:F in ratio 8:1] with theoretical Tt of 18°C, insoluble at *N. benthamiana* growing conditions was used in this work. Other synthetic ELPs expressed in plants have VPGVG repeat motif found which is less hydrophobic and has higher Tt (Floss et al., 2010). As was shown here, ELP[V8F1] induced PB formation and increased yields of TG2. Several reports have informed the effect of the fusion of ELP tag to foreign proteins in recombinant protein yields (Floss et al., 2010), but the impact of co-expression of ELP not fused to the protein of interest has scarcely been studied. Here, we showed that the number and size of PBs are increased by ELP[V8F1] co-expression and that induced PBs are heterogeneous since co-localization of ER-RFP-TG2 and ER-GFP was complete in the cortical region, but partial in the nuclear region of the cells. The existence of PBs with distinct composition could be atributed to different PB dynamics taking into account that PBs are highly mobile organelles dependent on actomyosin motility system (Conley et al., 2009, 2011). We showed that co-expression of the hydrophobic ELP[V8F1] increased accumulation of ER-GFP, ER-TG2, and vac-TG2 in 2.0-, 2.1-, and 2.5-fold, respectively. Similar results were obtained for secretory versions of erythropoietin and human interleukin-10 co-infiltrated with GFP-ELP and GFP-Hydrophobin I construct (Saberianfar et al., 2015). However, sec-RFP accumulation levels were not statistically different in the absence and presence of ELP, although formation of PBs and partial retention of sec-RFP inside these organelles was observed. Taken together, our results indicate that the effect of ELP on protein accumulation is dependent on the nature of the protein of interest and its final destination in the cell. The combination of a subcellular targeting strategies and PB induction by ELP co-expression were sufficient to increase TG2 accumulation levels in transient expression assays to allow further purification of both vac-TG2 and ER-TG2 using metal ion affinity chromatography.

Celiac Disease has a high worldwide prevalence and is largely undiagnosed (Garnier-Lengline et al., 2015) since only 1 out of seven patients are actually diagnosed (Rubio-Tapia et al., 2009). In Argentina, the prevalence is as high as in central Europe (Gomez et al., 2001). No massive screening test is performed since available methods based on detection of TG2 autoantibodies are expensive. Human recombinant TG2 is required for high sensitivity and specificity tests since it has superior performance compared to the guinea pig TG2 (Sardy et al., 1999; Rostom et al., 2006). Human TG2 produced in *E. coli* or insect cells is sold at 1,100 and 1,155 Euro/mg, respectively^3^. One of the advantages of plant expression system is the low manufacturing cost compared to other expression platform (Tusé et al., 2014).Considering yields of 20 mg/kg and similar cost to the ones reported for other plant-produced proteins (Tusé et al., 2014) for ER-TG2 and vac-TG2, production of TG2 by transient expression in tobacco will be considerably more economic, which would make this antigen more accessible for the development of massive screening tests. Importantly in this work we demonstrated that both ER-TG2 and vac-TG2 were recognized by IgA from peripheral blood of CD patients, and therefore are useful antigens for CD diagnosis. Further studies are under design to scale production of plant TG2 and to develop massive local screening test.

## Author Contributions

VV designed and performed experiments and analyzed data. GA built initial ELP constructs, MB cloned TG2 gene. FC and SP designed experiments, analyzed data, and supervised the project. All the authors have contributed significantly to the design, execution, and discussion of the manuscript.

## Conflict of Interest Statement

The authors declare that the research was conducted in the absence of any commercial or financial relationships that could be construed as a potential conflict of interest.

## Footnotes

1 http://www.kazusa.or.jp/codon/cgi-bin/showcodon.cgi?species=4100

2 http://rsb.info.nih.gov/ij/

3 http://zedira.com/Transglutaminases/Transglutaminase-2_3

## Abbreviations

TG2: tissue transglutaminase 2
CD: Celiac disease
CLSM: confocal laser scanning microscopy
d.p.i.: days post-infiltration
ELP: elastin-like polymer
ER: endoplasmic reticulum
IgA: immunoglobulin A
IM: infiltration medium
GFP: green fluorescent protein
mAb: monoclonal antibody
RFP: monomeric red fluorescent protein
RLS: Rubisco large subunit
RT: room temperature
VSS: vacuolar sorting signal
CT-VSS: carboxyl-terminal VSS
ssVSS: sequence specific VSS
TSP: total soluble protein
