# Supplementary figures and images for: Production of the Main Celiac Disease Autoantigen by Transient Expression in Nicotiana benthamiana
Source: Front Plant Sci. 2015 Dec 1;6:1067. doi: 10.3389/fpls.2015.01067 (PMC4664624; doi:10.3389/fpls.2015.01067)

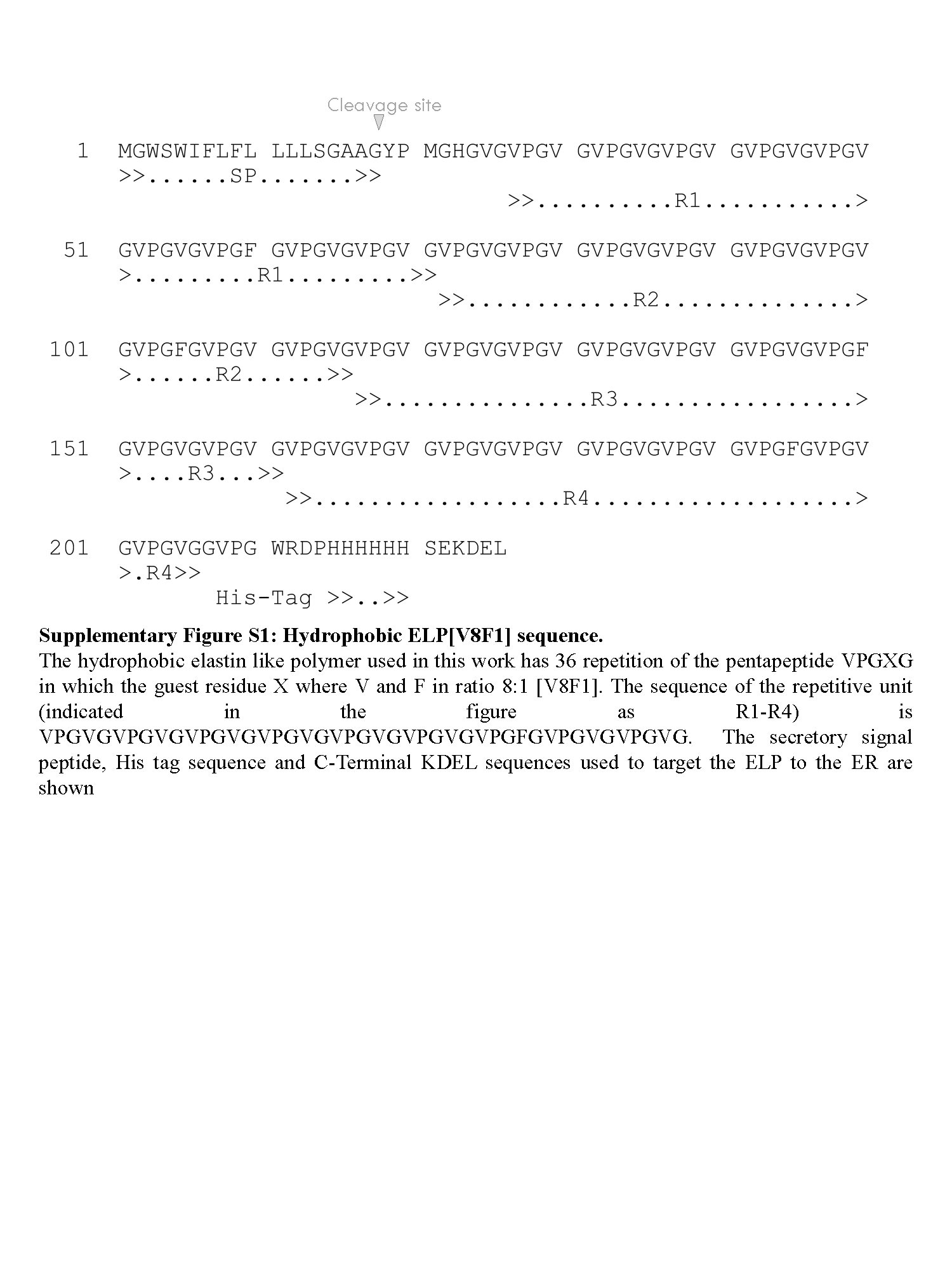

Supplement: Supplementary file 1 [file Image_1.TIFF]

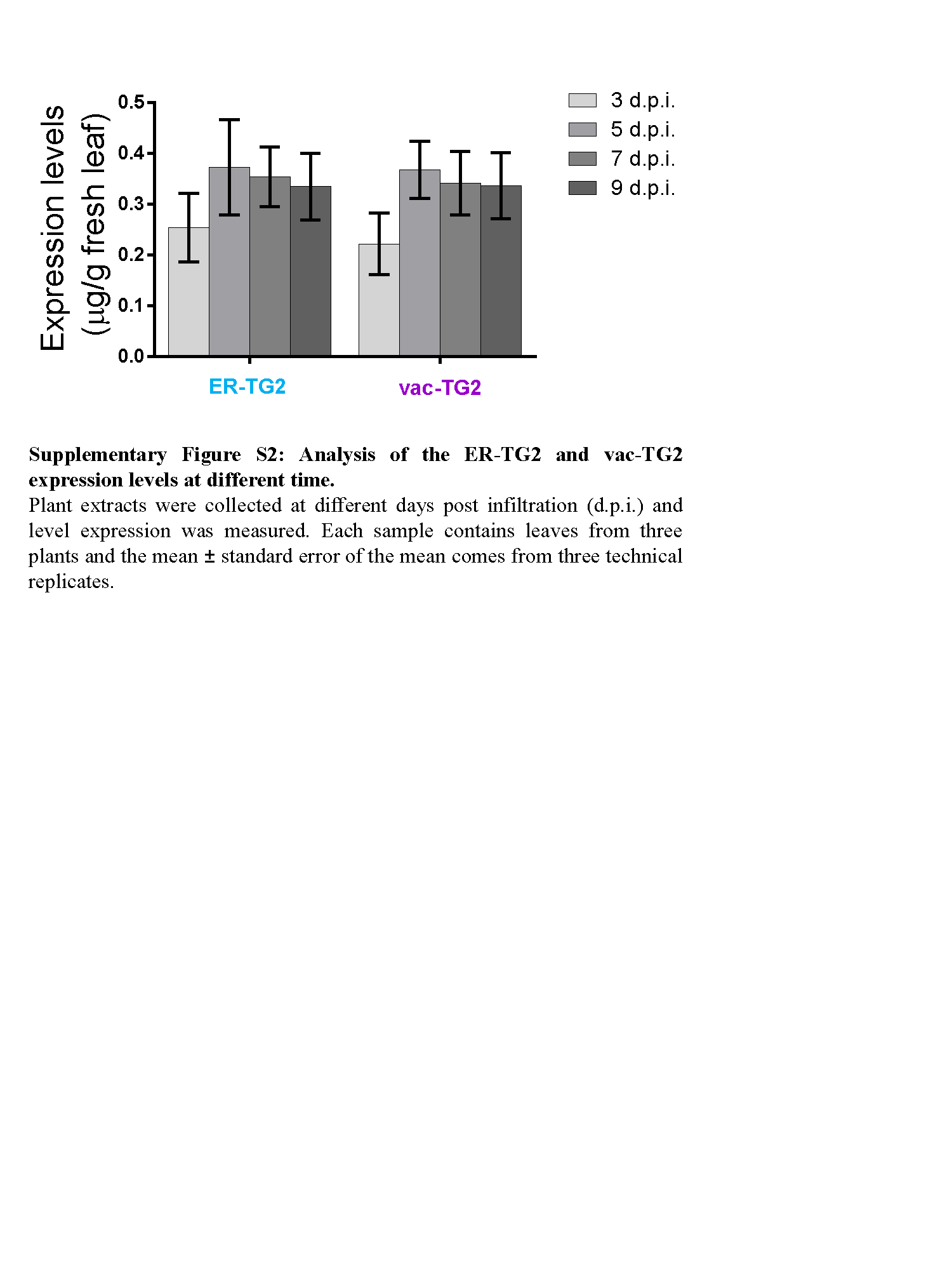

Supplement: Supplementary file 2 [file Image_2.TIFF]

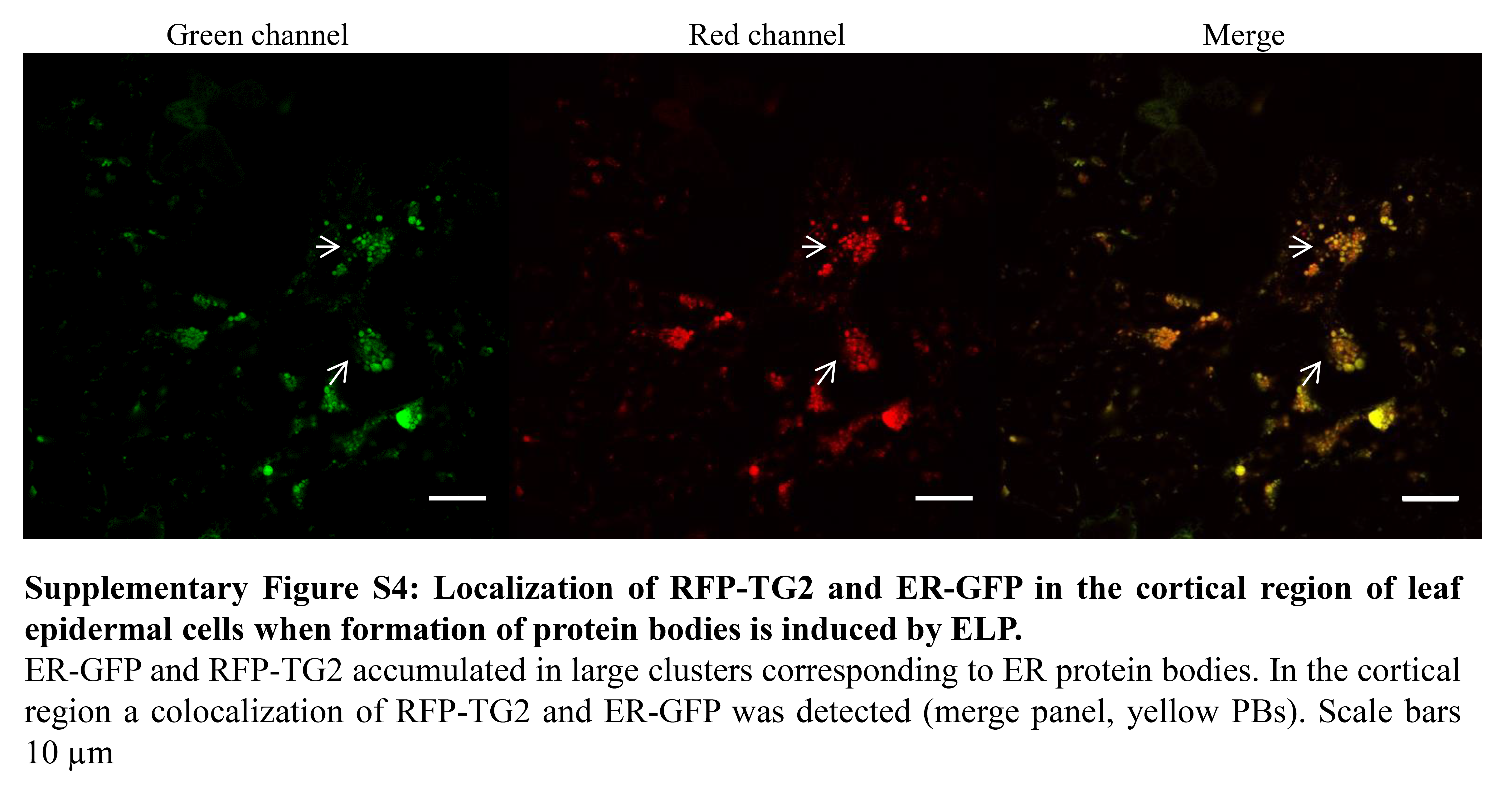

Supplement: Supplementary file 4 [file Image_4.TIF]

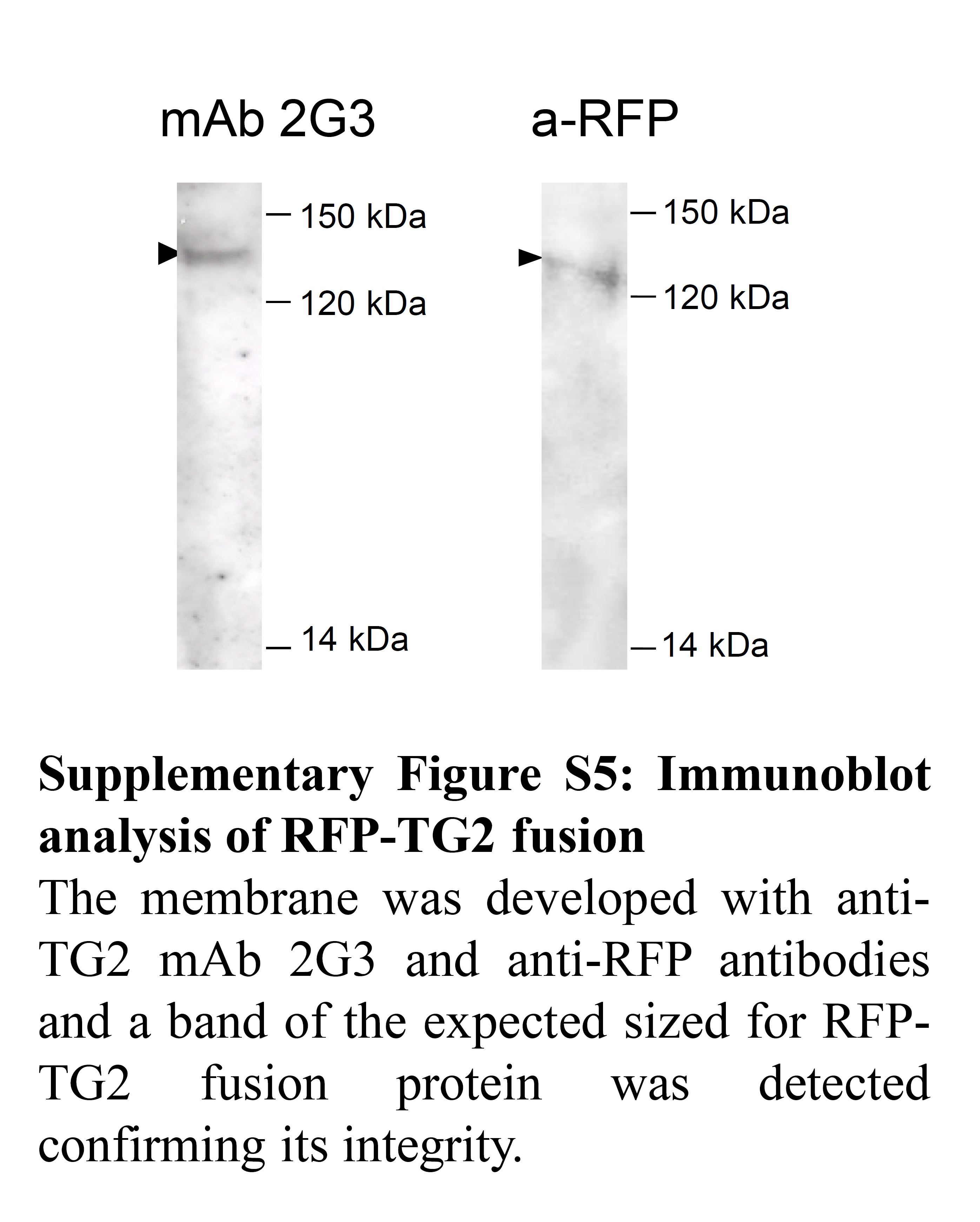

Supplement: Supplementary file 5 [file Image_5.TIF]
